# MicroRNA-1 Suppresses Tumor Progression and UHRF1 Expression in Cholangiocarcinoma

**DOI:** 10.3390/ijms262311718

**Published:** 2025-12-03

**Authors:** Makoto Muto, Teruhide Ishigame, Takashi Kimura, Naoya Sato, Yasuhide Kofunato, Akira Kenjo, Hiroyuki Yamamoto, Yuhki Yokoyama, Hirofumi Yamamoto, Shigeru Marubashi

**Affiliations:** 1 Department of Hepato-Biliary-Pancreatic and Transplant Surgery, Fukushima Medical University, 1 Hikarigaoka, Fukushima 960-1295, Japan; m-muto@fmu.ac.jp (M.M.); tkimura@fmu.ac.jp (T.K.); nawoya@fmu.ac.jp (N.S.); natty@fmu.ac.jp (Y.K.); a-kenjo@fmu.ac.jp (A.K.); s-maru@fmu.ac.jp (S.M.); 2 Department of Gastrointestinal Surgery, Aizu Medical Center, Fukushima Medical University, 21-2 Maeda, Yazawa, Kawahigashi-machi, Aizuwakamatsu 969-3492, Japan; 3 Department of Molecular Pathology, Division of Health Sciences, Graduate School of Medicine, Osaka University, 1-7 Yamadaoka, Suita 565-0871, Japan; otomamay@sahs.med.osaka-u.ac.jp (H.Y.); yyokoyama@sahs.med.osaka-u.ac.jp (Y.Y.); hyamamoto@sahs.med.osaka-u.ac.jp (H.Y.)

**Keywords:** cholangiocarcinoma, microRNA, miR-1, UHRF1, microarray

## Abstract

MicroRNAs (miRNAs) have been shown to suppress tumor progression in several cancers, including cholangiocarcinoma; however, their mechanisms and roles remain largely unexplored. In this study, we aimed to identify novel miRNAs and molecules associated with cholangiocarcinoma progression. Using miRNA and mRNA microarrays from surgically resected specimens, we identified microRNA-1 (miR-1) as significantly downregulated in cholangiocarcinoma tissues and selected it for further analysis. In the in vitro assay, miR-1 was transfected into cholangiocarcinoma cell lines to assess its effect on tumor progression. Proliferation and migration/invasion assays demonstrated significant tumor suppression in miR-1-transfected cells. Additionally, cell cycle and apoptosis assays revealed an increase in cells in the G1 and G2/M phases, a decrease in the S phase, and an elevation in apoptosis rates in miR-1-transfected cells. Further analysis of the miRNA and mRNA microarray data identified ubiquitin-like with plant homeodomain and ring finger domains 1 (UHRF1) as a molecule potentially associated with miR-1. Quantitative RT-PCR and Western blotting analysis confirmed that UHRF1 expression was suppressed following miR-1 transfection. This study demonstrated that cholangiocarcinoma progression was suppressed by restoring miR-1 expression, suggesting that UHRF1 expression is involved in cholangiocarcinoma progression. The results of this study provide new insights into the molecular mechanisms underlying cholangiocarcinoma progression.

## 1. Introduction

Cholangiocarcinoma (CCA) is a malignant tumor that develops in the epithelium of the intrahepatic or extrahepatic bile ducts. Surgery is the only radical treatment for CCA; however, the recurrence rate remains high.

The treatment options for recurrent or unresectable cases are limited, and chemotherapies using gemcitabine and cisplatin have been employed [1]. However, the effectiveness of these chemotherapies is insufficient to improve prognosis, with the 5-year overall survival rate after resection reported at 20–40% [2,3,4,5,6].

The widespread adoption of cancer genomic profiling tests has led to the discovery of actionable genomic mutations in CCAs, such as FGFR2, IDH1, BRAF, HER2, and NTRK [7]. However, the frequencies of these mutations are low, and treatment options are limited. Large-scale studies, such as The Cancer Genome Atlas (TCGA), have comprehensively analyzed genomic mutations, gene expression, protein expression, and miRNA expression across various cancers. However, data on CCA are extremely limited, with 40 and only 6 cases of intrahepatic and extrahepatic CCAs, respectively, in the TCGA database. Therefore, elucidating the mechanisms of tumor growth from novel perspectives and developing innovative therapeutics based on these insights are required.

MicroRNAs (miRNAs) are short-segment non-coding RNAs comprising approximately 22 nucleotides. miRNAs suppress mRNA expression by binding to corresponding seed sequences in the 3′UTR, inhibiting translation or reducing mRNA stability. This mechanism coordinates biological systems in healthy environments, and recent studies indicate that miRNAs are involved in cancer progression, including CCAs [8,9]. Oncogenic miRNAs promote cancer progression by inhibiting tumor suppressor genes and inducing metastasis by regulating genes associated with epithelial–mesenchymal transition. In contrast, tumor suppressor miRNAs regulate oncogenes and suppress cancer progression. miRNAs have attracted considerable attention and are being investigated as novel therapeutic targets.

In this study, we aimed to identify novel miRNAs and molecules associated with CCA progression.

## 2. Results

### 2.1. miR-1 Is Downregulated in CCA Tissue

**Figure 1:**
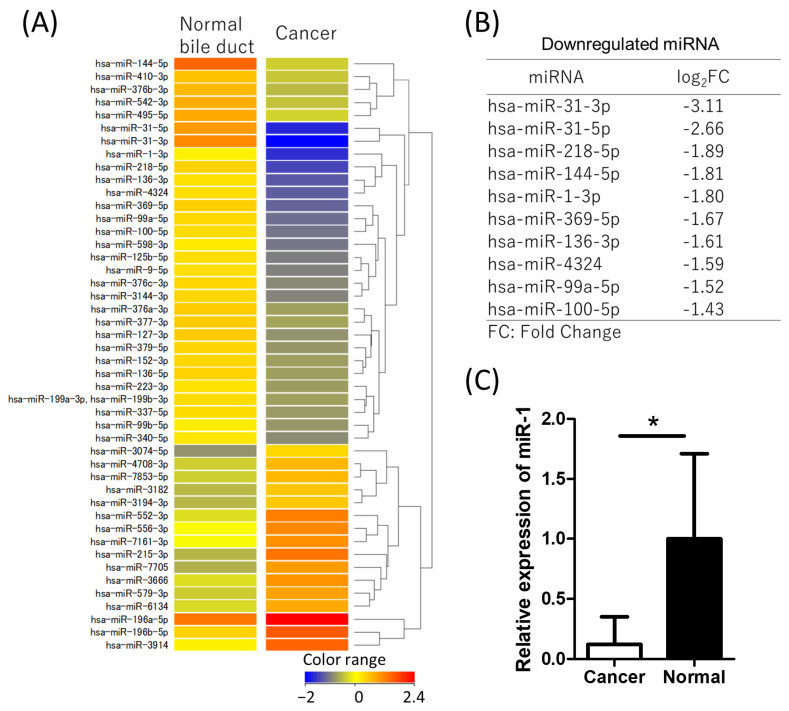
MicroRNA microarray and qRT-PCR of miR-1 of CCA tissue. Microarray data were normalized, and fold changes (FC) were calculated as the expression in cancer tissue relative to normal bile duct tissue. (**A**) MicroRNA microarray heatmap showed 30 downregulated miRNAs (log2FC < −1.0) and 16 upregulated miRNAs (log2FC > 1.0) in bile duct cancer. (**B**) The most downregulated miRNAs identified in the microarray are listed. (**C**) qRT-PCR validation shows that miR-1 expression is significantly downregulated in cancer tissue. * *p* < 0.001 compared to normal bile duct group.

miRNA and mRNA microarrays were performed to detect changes in RNA expression in CCA tissues compared with normal bile ducts obtained from surgically resected specimens. Four samples from CCA and normal bile ducts were analyzed. In the miRNA microarray, 30 miRNAs were downregulated (log2FC < −1.0), whereas 16 miRNAs were upregulated (log2FC > 1.0) (Figure 1A). Figure 1B presents the list of downregulated miRNAs with large folds. Among these downregulated miRNAs, miR-1 was selected for further investigation as a potential target because of the lack of previous reports on its role in CCA.

For validation, qRT-PCR was performed on other samples used for the microarray analysis to evaluate miR-1 expression (*n* = 4 for CCA and normal bile ducts). qRT-PCR analysis revealed that the relative expression of miR-1 was significantly downregulated (Figure 1C). These results indicate that miR-1 is downregulated in CCA, prompting further investigation into its role in this study.

### 2.2. miR-1 Suppresses Cell Proliferation, Migration, Invasion, and Cell Cycle Progression, While Promoting Apoptosis in CCA Cell Lines

**Figure 2:**
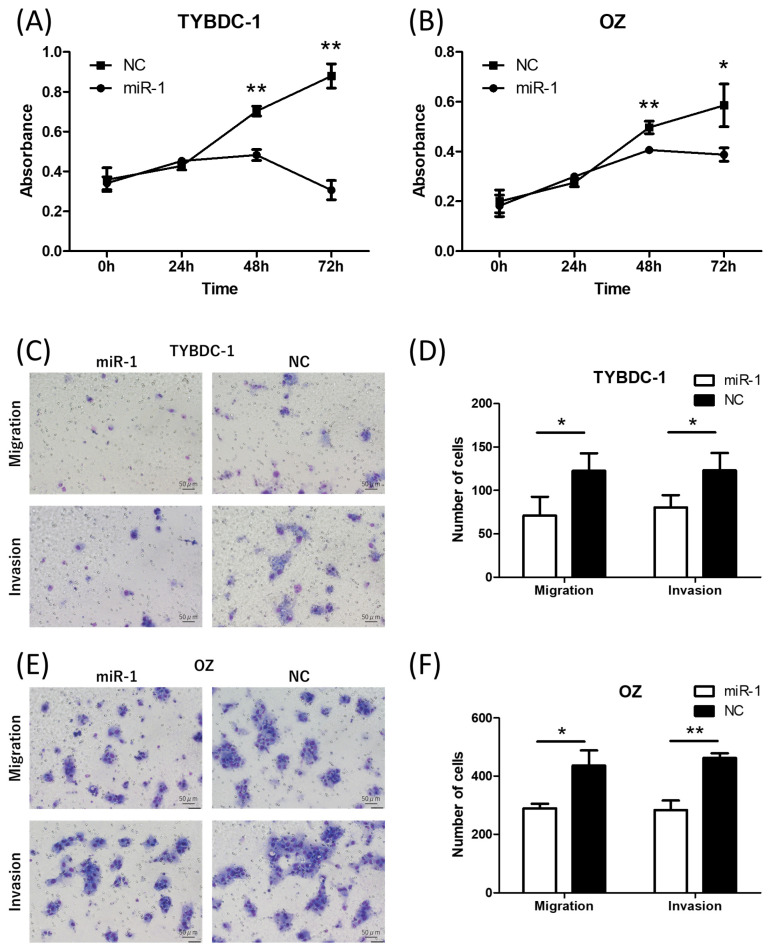
Proliferation assay and migration/invasion assay of CCA cell lines by miR-1 transfection. (**A**,**B**) Cell proliferation was suppressed by miR-1 transfection in TYBDC-1 and OZ cells at 48 and 72 h, respectively. (**C**–**F**) Cell migration and invasion were suppressed by miR-1 transfection in TYBDC-1 and OZ cells. * *p* < 0.05 and ** *p* < 0.001 compared to negative control (NC).

Proliferation, migration/invasion, cell cycle, and apoptosis assays were performed to investigate the effects of miR-1 on CCA progression. miR-1 and negative control mimics were transfected into TYBDC-1 and OZ cells in all assays, as described above. The proliferation assay revealed that miR-1 transfection suppressed cell proliferation in TYBDC-1 and OZ cells at 48 and 72 h, respectively (Figure 2A,B and Figure S1).

In the migration and invasion assays, transfected cells were analyzed 48 h after cell seeding using a Transwell permeable support. The results indicated that miR-1 transfection suppressed cell migration and invasion in TYBDC-1 and OZ cells (Figure 2C–F).

**Figure 3:**
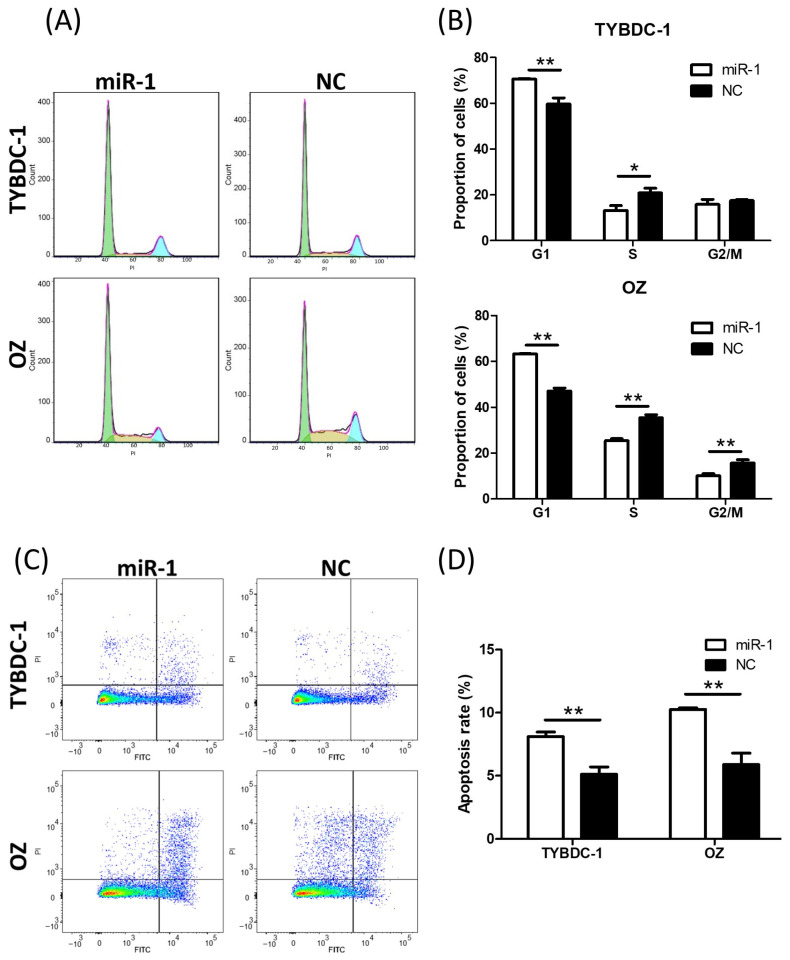
Cell cycle and apoptosis assay of CCA cell lines by miR-1 transfection. (**A**,**B**) Cell cycle analysis shows an increase in the proportion of cells in the G1 and G2/M phases, and a decrease in the S phase in miR-1 transfected cells. Green indicates the G1 phase, yellow indicates the S phase, and blue indicates the G2/M phase. (**C**,**D**) Apoptosis assays reveal a significant increase in apoptosis rates in miR-1 transfected cells. * *p* < 0.05 and ** *p* < 0.001 compared to negative control (NC).

In the cell cycle assay, cells were analyzed 24 h after transfection, and the proportions of cells in the G1, S, and G2/M phases were assessed. The G1 and G2/M phases increased, whereas the S phase decreased in miR-1 transfected cells (Figure 3A,B). These results indicate that miR-1 suppresses DNA replication and mitosis.

In the apoptosis assay, transfected cells were analyzed 48 h after transfection, and the apoptosis rate increased in miR-1 transfected cells (Figure 3C,D). These results suggest that miR-1 suppressed tumor progression in CCA.

### 2.3. UHRF1 Expression Is Suppressed by miR-1 Transfection

To identify potential targets of miR-1, pathway analysis was conducted using Ingenuity Pathway Analysis (IPA) based on the miRNA and mRNA microarray data. Pathway analysis predicted five possible miR-1 target genes, including ubiquitin-like with plant homeodomain and ring finger domains 1 (UHRF1), KRAS, neuropilin and tolloid-like 2 (NETO2), agmatinase (AGMAT), and chromosome 17 open reading frame 78 (C17orf78) (Figure S2).

**Figure 4:**
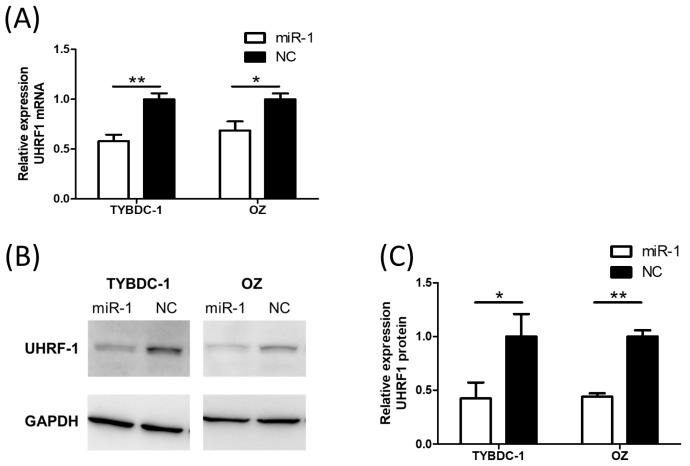
UHRF1 expression analysis by RT-qPCR and Western blotting in CCA cell lines by miR-1 transfection. (**A**) miR-1 transfection significantly reduces UHRF1 mRNA expression. (**B**,**C**) Western blot analysis shows a reduction in UHRF1 protein levels following miR-1 transfection. * *p* < 0.05 and ** *p* < 0.001 compared to negative control (NC).

Among these five genes, UHRF1 was selected for further investigation because of its established role in cancer progression. qRT-PCR was performed to explore the changes in UHRF1 mRNA expression caused by miR-1 transfection. UHRF1 expression was suppressed in miR-1 transfected cells (Figure 4A). Western blot analysis was performed to evaluate the changes in protein levels. UHRF1 protein expression was suppressed by miR-1 transfection (Figure 4B,C). These results demonstrated that miR-1 downregulates UHRF1 at the mRNA and protein levels.

## 3. Discussion

In this study, we demonstrated that miR-1 suppresses CCA progression in vitro. Microarray-based screening of miRNA and mRNA expression identified miR-1 as one of the most significantly downregulated miRNAs in CCA tissues, prompting its selection for further functional analysis. Transfection of miR-1 into CCA cell lines inhibited cell proliferation, migration, and invasion, while also inducing cell-cycle arrest and apoptosis. Through pathway analysis, UHRF1 emerged as a potential downstream target of miR-1, and miR-1 overexpression markedly reduced UHRF1 expression at both the mRNA and protein levels.

miRNAs are short, non-coding RNA segments that regulate gene expression by inhibiting mRNA translation or reducing mRNA stability. This regulatory mechanism is closely associated with tumor progression, with several studies demonstrating relationships between miRNAs and various cancers. In CCA, miRNAs such as miR-96, miR-129, miR-137, miR-144, miR-193, miR-373, and miR-551 have been identified to promote or suppress tumor progression [10,11,12,13,14,15,16].

The role of miR-1 has been documented in various cancers, including ovarian, breast, lung, prostate, bladder, renal, esophageal, gastric, hepatic, colorectal, and thyroid cancers [17,18,19,20,21,22,23,24,25,26,27]. In these cancers, miR-1 inhibits tumor progression by suppressing specific target genes. For instance, in small-cell lung cancer, miR-1 directly suppresses C-X-C motif chemokine receptor 4 (CXCR4), leading to the subsequent inhibition of FOXM1 and ribonucleotide reductase M2 (RRM2), thereby impeding tumor progression [27]. Similarly, in bladder cancer, miR-1 mediates tumor suppression by directly targeting Golgi phosphoprotein 3 (GOLPH3), inhibiting FOXO1 and AKT phosphorylation [23]. Other molecules, including dynein light chain Tctex-type 3 (DYNLT3), PFTAIRE protein kinase 1 (PFTK1), E2F5, Fibronectin1, centromere protein F (CENPF), apoptosis inhibitor-5 (API-5), polypyrimidine tract-binding protein 1 (PTBP1), and HOX antisense transcript RNA (HOTAIR), have been identified as targets of miR-1. However, the role of miR-1 in CCA has not been previously reported.

UHRF1 is an epigenetic gene regulator that silences tumor suppressor genes by regulating DNA methylation in cancer cells [28]. DNA methylation occurs at cytosines within cytosine–phosphate–guanine (CpG) dinucleotides and plays a crucial role in suppressing gene expression. CpG islands, which are regions with high densities of CpG dinucleotides, are often located in promoter regions of genes. Methylation of these regions inhibits the binding of transcription factors, suppressing gene expression. UHRF1 is essential for maintaining methylation patterns on newly synthesized DNA strands during the S phase of replication, thereby silencing tumor suppressor genes. Additionally, UHRF1 is involved in histone modification, another important epigenetic mechanism. UHRF1 interacts with specific regions of histones, such as di- and tri-methylated lysine 9 of histone H3 (H3K9me2/3) and unmodified arginine 2 of histone H3 (H3R2), and its role in histone methylation and deacetylation results in the inhibition of gene expression. In various cancers, including bladder, colorectal, renal, lung, and gastric cancers, the tumor-suppressive regulation of UHRF1 by miRNAs has been reported. Specifically, miR-9, miR-101, miR-124, miR-145, miR-146a/b, and miR-193a directly bind to the 3′UTR of UHRF1, thereby suppressing its expression [29,30,31,32,33,34,35]. Additionally, one report indicated that miR-124 suppresses UHRF1 expression in intrahepatic CCA, a type of biliary tract cancer [36].

UHRF1 suppresses the expression of tumor-suppressor genes by promoting methylation of their promoters [37]. In breast cancer, BRCA1 has been reported as a target of UHRF1-mediated methylation, while in non-small-cell lung cancer, RASSF1, CYGB, and CDH13 have been identified as UHRF1-regulated methylation targets. In colorectal cancer and other malignancies, p16 has been suggested as a potential target. In CCA, hypermethylation of the promoters of RASSF1, p16, and CDH13 has been documented, raising the possibility that these genes may also be regulated by alterations in miR-1 and UHRF1 expression [38,39]. Investigating whether these molecules are epigenetically modulated through the miR-1–UHRF1 axis will be essential for elucidating the epigenetic mechanisms underlying tumor regulation mediated by miR-1 and UHRF1.

Pathway analysis from our microarray data identified several potential miR-1 target genes, including UHRF1, KRAS, NETO2, AGMAT, and C17orf78. Among these candidates, we selected UHRF1 as a molecule for detailed investigation by considering both the novelty of the research focus and the likelihood that each gene represents a direct target of miR-1. KRAS is a well-established oncogenic driver whose activating mutations constitutively stimulate the RAF/MEK/ERK and phosphoinositide 3-kinase (PI3K)/AKT/mTOR pathways, thereby promoting uncontrolled proliferation, differentiation abnormalities, drug resistance, and remodeling of the tumor microenvironment [40]. Direct binding and repression of KRAS by miR-1 have been demonstrated in several tumor types, suggesting that KRAS may also contribute to miR-1-mediated tumor suppression in CCA [41,42]. NETO2, initially characterized as a kainate-receptor-associated transmembrane protein in neuronal biology, has recently emerged as an oncogenic factor in gastrointestinal and thoracic malignancies [43]. Upregulation of NETO2 enhances cancer progression by activating PI3K/AKT- and ERK/MAPK-dependent cascades and downstream transcription factors such as NF-κB, Snail, and Nrf2, thereby promoting epithelial–mesenchymal transition, proliferation, migration, and invasion [44,45]. Although miR-1 has not yet been reported as a regulator of NETO2, its 3′UTR contains putative miR-1 binding sites, and our preliminary qRT-PCR experiments demonstrated miR-1-dependent suppression of NETO2 and KRAS transcripts. In contrast, AGMAT and C17orf78 lack seed-matching sequences within their 3′UTRs and are unlikely to be direct miR-1 targets. UHRF1 harbors only a 6-mer seed match for miR-1 within its 3′UTR, suggesting that any direct interaction would be relatively weak. Nevertheless, the potential for an uncharacterized regulatory relationship, combined with extensive evidence implicating UHRF1 in tumorigenesis across multiple cancers, made UHRF1 a biologically compelling candidate for functional investigation in CCA.

To clarify the mechanism by which miR-1 regulates UHRF1, we performed a luciferase reporter assay; however, direct binding of miR-1 to the 3′UTR of UHRF1 could not be demonstrated. Given that a 6-mer seed match typically provides insufficient affinity for stable repression compared with canonical 7–8-mer matches, the likelihood of a strong direct interaction is low. These findings suggest that UHRF1 repression by miR-1 is more likely mediated through an indirect regulatory mechanism. We therefore considered cyclin-dependent kinase 4/6 (CDK4/CDK6) as plausible intermediaries [37]. CDK4/6 possess high-affinity 7-mer and 8-mer seed matches for miR-1, and they are upstream regulators of UHRF1 transcription through the RB/E2F pathway. miR-1-mediated suppression of CDK4/CDK6 may reduce RB phosphorylation, attenuate E2F1 activation, and consequently downregulate UHRF1 expression. This proposed mechanism aligns with our observation that miR-1 substantially reduces UHRF1 levels despite the lack of demonstrable direct 3′UTR binding.

In the context of these findings, enhancing miR-1 delivery and stability may be crucial for modulating downstream epigenetic regulators such as UHRF1. However, the clinical translation of miRNA-based therapeutics remains limited by rapid nuclease degradation, suboptimal cellular uptake, and non-specific accumulation in normal tissues. Several delivery systems have been developed to address these challenges, including lipid-based nanoparticles, polymeric vectors, and inorganic carriers such as carbonate apatite, which improve molecular stability and facilitate endosomal escape while minimizing immunogenicity [9,46]. Chemical modification of miRNAs—including ligand conjugation and backbone/ribose stabilization—has further enhanced serum stability and improved biodistribution without relying on complex viral vector systems [47]. These technological advances collectively support the feasibility of developing miRNA-based therapies and may augment the therapeutic potential of miR-1 to suppress UHRF1-mediated epigenetic dysregulation in CCA.

The clinical relevance of miR-1 and UHRF1 in CCA remains insufficiently understood. Although a few studies have suggested that UHRF1 overexpression may serve as an adverse prognostic factor in intrahepatic CCA, our analysis of the TCGA-CHOL cohort (approximately 40 cases) did not reveal a clear association between the expression levels of miR-1 or UHRF1 and patient survival (Figure S3) [36]. Given the rarity of CCA and the limited size of available datasets, larger patient cohorts will be required to more definitively determine the clinicopathological significance of the miR-1–UHRF1 axis.

A limitation of this study is the inability to obtain consistent rescue effects by UHRF1 overexpression in miR-1–transfected CCA cells, possibly due to technical difficulties with double transfection. More stable expression systems will be necessary to determine whether UHRF1 functions as a critical downstream mediator of miR-1. Although direct binding between miR-1 and the 3′UTR of UHRF1 was not demonstrated, our data support an indirect regulatory mechanism that warrants further validation. Together, these findings provide new insight into the miR-1/UHRF1 axis in CCA and highlight the need for additional mechanistic and translational studies.

## 4. Materials and Method

### 4.1. Clinical Tissue Samples

CCA and normal bile duct tissues were obtained from 19 patients who underwent surgery for CCA at Fukushima Medical University Hospital (Fukushima, Japan). Obtained tissues were frozen in liquid nitrogen and stored at −80 °C. The Ethics Committee of Fukushima Medical University approved this study, and informed consent was obtained from all patients.

### 4.2. miRNA and mRNA Microarray Analysis

miRNA and mRNA microarray analyses were performed to determine the expression levels of CCA.

RNA extraction from frozen tissues and microarray procedures were performed by Toray Industries Inc. (Tokyo, Japan) using a 3D-Gene Human miRNA Oligo Chip (miRBase version 21) and Human Oligo Chip 25k.

Microarray data were analyzed using GeneSpring GX (Agilent Technologies, Santa Clara, CA, USA). Signal intensities were normalized using the global normalization method and expressed as binary logarithms. Each of the four samples was analyzed for CCA and normal bile duct, and the fold changes in RNA expression level were calculated.

### 4.3. Cell Culture and Transfection

TYBDC-1 and OZ, human CCA cell lines, were purchased from the Japanese Collection of Research Bioresources Cell Bank, National Institutes of Biomedical Innovation, Health and Nutrition (Osaka, Japan). TYBDC-1 cells were cultured in DMEM/F-12 (Gibco; Thermo Fisher Scientific Inc., Waltham, MA, USA) and OZ cell was cultured in William’s E medium (Gibco), with each medium containing 10% FBS (NICHIREI BIOSCIENCES Inc., Tokyo, Japan) and 1% penicillin–streptomycin solution (Sigma-Aldrich, St. Louis, MO, USA). Cell lines were cultured in an incubator at 5% CO2 and 37 °C.

mirVana miR-1-3p mimic (Cat#4464066) and mirVana miRNA mimic Negative Control #1 (Cat#4464058) were obtained from Invitrogen (Thermo Fisher Scientific Inc., Waltham, MA, USA), and miRNA mimics were transfected into cell lines using Lipofectamine RNAiMAX Reagent (Invitrogen, Thermo Fisher Scientific Inc., Waltham, MA, USA) according to the manufacturer’s protocol. All transfection experiments were performed in triplicate.

### 4.4. RNA Extraction and Quantitative RT-PCR (qRT-PCR)

TYBDC-1 and OZ cells were seeded at a density of 0.3 × 10^6^/well in 6-well plates, and miR-1 and negative control mimic (TYBDC-1: 30 nM, OZ: 30 nM) were transfected 48 h after cell seeding. Transfected cells were lysed 24 h after transfection using TRIzol reagent (Invitrogen) according to the manufacturer’s protocol.

For the qRT-PCR of miRNAs, reverse transcription to cDNA was performed using TaqMan MicroRNA Reverse Transcription Kit (Applied Biosystems; Thermo Fisher Scientific Inc., Waltham, MA, USA), followed by PCR using TaqMan Universal Master Mix II, with UNG (Applied Biosystems).

For qRT-PCR of mRNAs, reverse transcription to cDNA was performed using SuperScript Ⅳ VILO Master Mix with ezDNase Enzyme (Invitrogen), and PCR was performed using TaqMan Fast Advanced Master Mix (Applied Biosystems).

The StepOnePlus Real-Time PCR System (Applied Biosystems) was used for qRT-PCR reactions according to the manufacturer’s protocol. Assays were performed in triplicate. U6 for miRNA and GAPDH for mRNA were used for internal control reference, and relative expression was calculated using the 2^−ΔΔCt^ method. The following TaqMan MicroRNA Assays and TaqMan Gene Expression Assays (Applied Biosystems) were used: miR-1-3p (Cat#4427975, Assay ID 002222), U6 snRNA (Cat#4427975, Assay ID 001973), ubiquitin-like with plant homeodomain and ring finger domains 1 (UHRF1; Cat#4331182, Assay ID Hs01086727_m1), and GAPDH (Cat#4331182, Assay ID Hs02786624_g1).

### 4.5. Proliferation Assay

Cell proliferation was assessed using CCK-8 (Dojindo Laboratories, Kumamoto, Japan). TYBDC-1 and OZ cells were seeded at a density of 5000/well in 96-well plates. miR-1 and negative control mimics were transfected 24 h after cell seeding (TYBDC-1: 10 nM, OZ: 30 nM). At 0, 24, 48, and 72 h after transfection, a CCK-8 reagent of 10 ul was added to each well. After 2 h of incubation, the absorbance at 450 nm was measured using Multidkan GO (Thermo Fisher Scientific Inc.).

### 4.6. Migration and Invasion Assay

Cell migration and invasion abilities were assessed with 24-well Transwell permeable support (polycarbonate membrane, 8 μm pore size, Corning, NY, USA). The upper chamber membrane of was coated with Corning Matrigel basement membrane matrix (Corning) for the invasion assay. TYBDC-1 and OZ cells were seeded at a density of 0.5 × 10^6^/well in 6-well plates, and miR-1 and negative control mimics were transfected 48 h later (TYBDC-1: 10 nM, OZ: 30 nM).

After 24 h, the cells were collected, and 2.0 × 10^6^ cells resuspended in 200 µL of serum-free medium were seeded into the upper chamber of a Transwell permeable support, with 700 μL of culture medium containing 10% FBS added to the lower chamber. After 48 h, the membranes of the upper chamber were stained with Diff-Quik (Sysmex, Kobe, Japan) according to the manufacturer’s protocol. Migrating and invading cells through the membrane were counted using a microscope in five fields at ×200 magnification in triplicate.

### 4.7. Cell Cycle Assay

TYBDC-1 and OZ cells were seeded at a density of 0.3 × 10^6^/well with culture medium containing 10% FBS in 6-well plates and cells were starved in serum-free medium 24 h after cell seeding. Cell starvation continued for 48 h and miR-1 and negative control mimics were transfected (TYBDC-1: 10 nM, OZ: 30 nM) 24 h before the end of starvation. The transfected cells were collected 24 h after starvation and fixed in 70% ethanol. The fixed cells were washed with PBS, and FxCycle PI/RNase Staining Solution (Invitrogen) was added. After 30 min of incubation, the cells were analyzed by flow cytometry using a BD FACSCanto II (BD Biosciences, San Jose, CA, USA).

### 4.8. Apoptosis Assay

The apoptosis assay was conducted using the Annexin V Apoptosis Detection Kit FITC (Invitrogen) and analyzed via flow cytometry. TYBDC-1 and OZ cells were seeded at a density of 0.3 × 10^6^/well in 6-well plates, and transfected with miR-1 and negative control mimics (TYBDC-1: 10 nM, OZ: 30 nM) 48 h after cell seeding. Transfected cells were collected 48 h after transfection. Annexin V and PI staining solutions were added to the collected cells, followed by analysis via flow cytometry using a BD FACSCanto II (BD Biosciences).

### 4.9. Prediction of miRNA Target Genes

Ingenuity Pathway Analysis (IPA; Qiagen, Germantown, MD, USA) was used to predict miRNA target genes using miRNA and mRNA microarray data through the web-based platform (analysis performed using the version available in 2020). IPA predicted the target genes of miRNAs based on the miRNA-mRNA expression pairing with miRNA databases, including TargetScan Human, TarBase, and miRecords.

### 4.10. Western Blotting Analysis

TYBDC-1 and OZ cells were seeded at a density of 0.3 × 10^6^/well in 6-well plates, and transfected with miR-1 and negative control mimics (TYBDC-1:50 nM, OZ:50 nM) 48 h after cell seeding. Transfected cells were lysed with RIPA Lysis and Extraction Buffer (Thermo Scientific: Thermo Fisher Scientific Inc., Waltham, MA, USA) 48 h after transfection. Protein lysates were electrophoresed using SDS-PAGE on a 4–20% polyacrylamide gel and transferred to a PVDF membrane (Invitrogen). Membranes were incubated overnight at 4 °C with primary antibody against UHRF1 (SAB1405050, Sigma-Aldrich), and at room temperature for 1 h with GAPDH (G8795, Sigma-Aldrich). HRP-conjugated Goat Anti-Mouse IgG antibody (ab6789, Abcam, Cambridge, UK) was used as the secondary antibody, and membranes were incubated at room temperature for 1 h. The bands were visualized using ECL with SuperSignal West Pico PLUS Chemiluminescent Substrate (Thermo Scientific). Protein expression levels were analyzed using ImageJ software (version 1.53a; National Institutes of Health, Bethesda, MD, USA) and normalized to GAPDH.

### 4.11. Statistical Analysis

Experimental data are expressed as mean ± SD, and statistical analyses were performed using SPSS version 26 (IBM Corp., Armonk, NY, USA). For qRT-PCR analysis, data were assessed using the 2^−ΔΔCt^ method and presented as mean with range calculated by the SD of ΔΔCt values. Student’s *t*-test was used to compare differences between the two groups, with a *p*-value < 0.05 considered statistically significant.

## 5. Conclusions

In conclusion, our study demonstrated the tumor-suppressive role of miR-1 and its ability to suppress UHRF1 expression in CCA. These findings are important for advancing our understanding of the mechanisms underlying CCA progression; however, further research is required to fully elucidate these processes.

## Data Availability

The raw data supporting the conclusions of this article will be made available by the authors on request.

## Supplementary Materials

The following supporting information can be downloaded at: https://www.mdpi.com/article/10.3390/ijms262311718/s1.

## Abbreviations

The following abbreviations are used in this manuscript:

3′UTR	3′ untranslated region
AGMAT	agmatinase
API-5	apoptosis inhibitor-5
CCA	cholangiocarcinoma
CDH13	cadherin 13
CDK4/CDK6	cyclin-dependent kinase 4/6
CENPF	centromere Protein F
C17orf78	chromosome 17 open reading frame 78
CpG	cytosine–phosphate–guanine
CXCR4	C-X-C motif chemokine receptor 4
DYNLT3	dynein light chain Tctex-type 3
GOLPH3	Golgi phosphoprotein 3
H3K9me2/3	di- and tri-methylated lysine 9 of histone H3
H3R2	unmodified arginine 2 of histone H3
HOTAIR	HOX antisense transcript RNA
IPA	Ingenuity Pathway Analysis
miRNA	microRNA
miR-1	microRNA-1
NETO2	neuropilin and tolloid-like 2
NF-kB	nuclear factor kappa-light-chain-enhancer of activated B cells
PFTK1	PFTAIRE protein kinase 1
PI3K	phosphoinositide 3-kinase
PTBP1	polypyrimidine tract-binding protein 1
qRT-PCR	quantitative RT-PCR
RASSF1	Ras association domain family member 1
RRM2	ribonucleotide reductase M2
TCGA	The Cancer Genome Atlas
UHRF1	ubiquitin-like with plant homeodomain and ring finger domains 1
