# Supplementary material for: MicroRNA-1 Suppresses Tumor Progression and UHRF1 Expression in Cholangiocarcinoma
Source: Int J Mol Sci. 2025 Dec 3;26(23):11718. doi: 10.3390/ijms262311718 (PMC12692032; doi:10.3390/ijms262311718)
Supplement: Supplementary file 1 [file ijms-26-11718-s001.zip › ijms-3994304-supplementary.pdf]

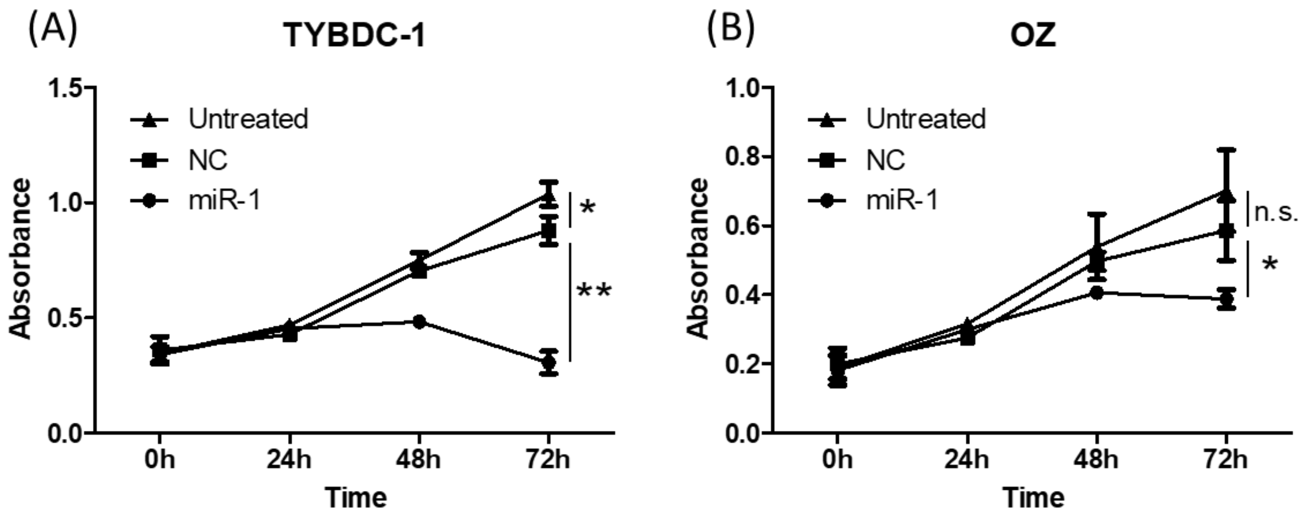

Figure S1

Proliferation assay of CCA cell lines following miR-1 transfection, including untreated cells.

(A) (B) Cell proliferation was suppressed by miR-1 transfection in TYBDC-1 and OZ cells at 48 and 72 hours, respectively. Although the negative control miRNA showed a modest reduction compared with the untreated control, the extent of this decrease remained limited. *$p < 0.05$, **$p < 0.001$, n.s.: not significant.

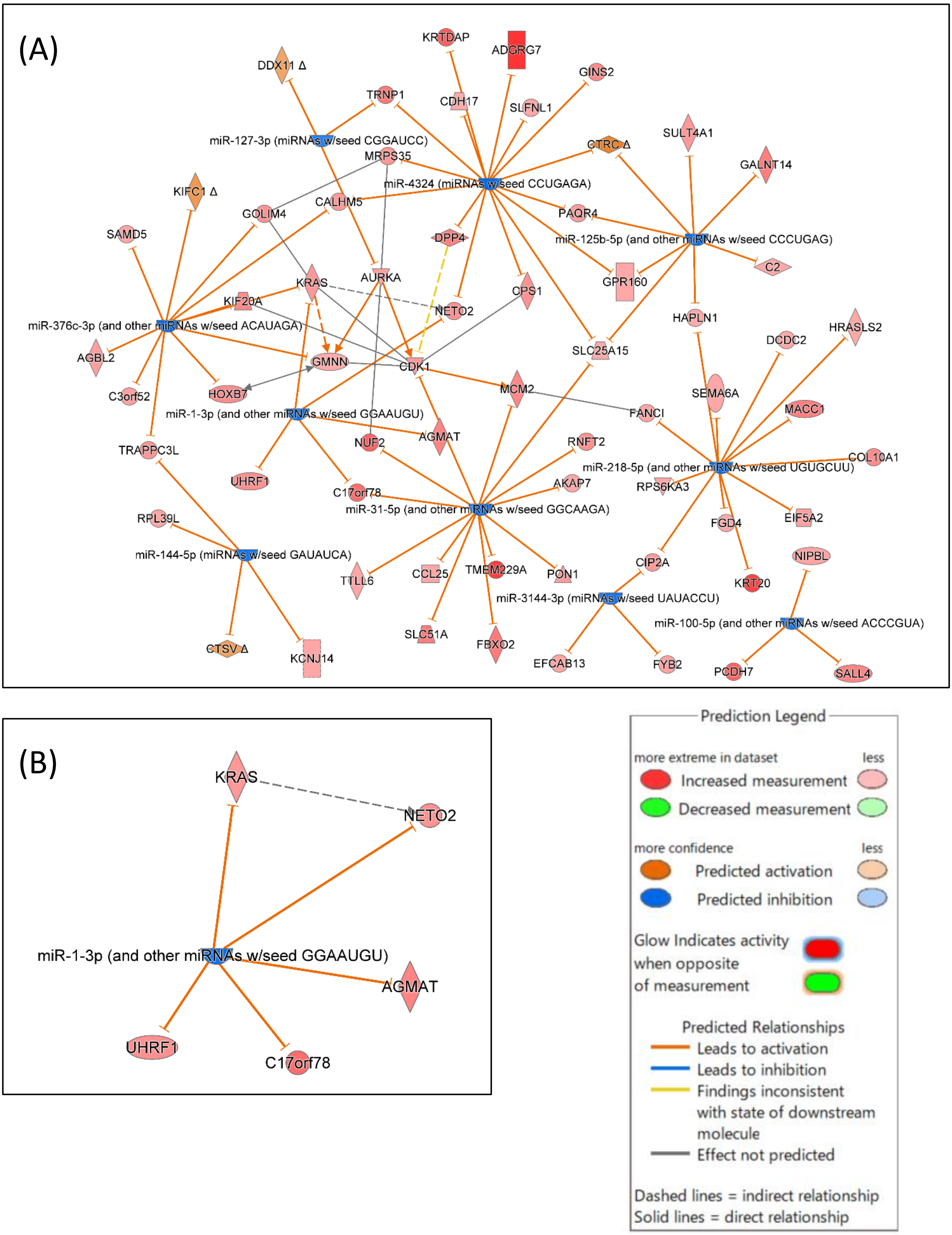

Figure S2

Potential target genes of miRNAs predicted by IPA based on miRNA and mRNA microarray data.

(A) Ten miRNAs downregulated in CCA and their potential target genes.

(B) MiR-1 and its potential target genes (UHRF1, KRAS, NETO2, AGMAT, and C17orf78).

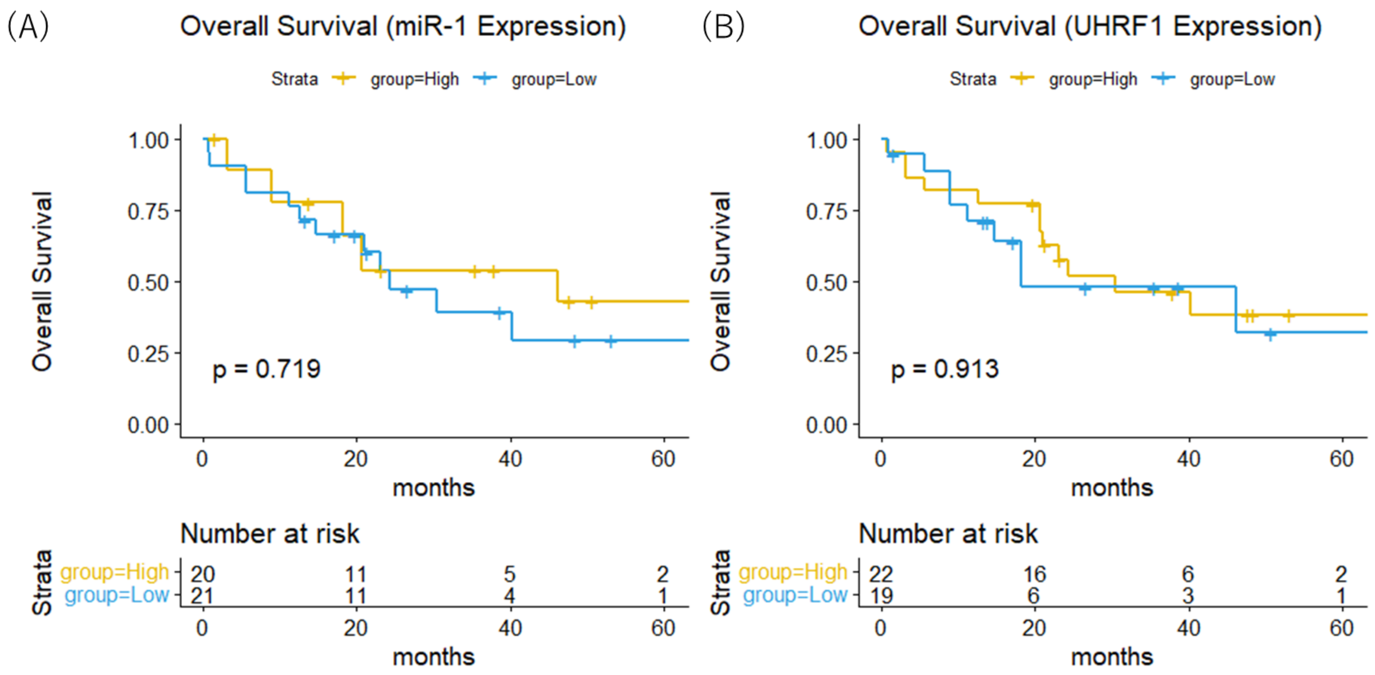

Figure S3

Survival analysis of CCA using the TCGA-CHOL dataset.

(A) (B) Overall survival stratified by miR-1 and UHRF1 expression showed no significant differences.
